# Transient disease dynamics across ecological scales

**DOI:** 10.1007/s12080-021-00514-w

**Published:** 2021-05-27

**Authors:** Yun Tao, Jessica L. Hite, Kevin D. Lafferty, David J. D. Earn, Nita Bharti

**Affiliations:** 1 Intelligence Community Postdoctoral Research Fellowship Program, Department of Ecology, Evolution and Marine Biology, University of California, Santa Barbara, CA 93106 USA; 2 School of Veterinary Medicine, Department of Pathobiological Sciences, University of Wisconsin, Madison, WI 53706 USA; 3 Western Ecological Research Center at UCSB Marine Science Institute, U.S. Geological Survey, CA 93106 Santa Barbara, USA; 4 Department of Mathematics and Statistics, McMaster University, Hamilton, ON L8S 4K1 Canada; 5 Department of Biology Center for Infectious Disease Dynamics, Penn State University, University Park, PA 16802 USA

**Keywords:** Transients, Disease ecology, Epidemiology, Dynamical systems

## Abstract

Analyses of transient dynamics are critical to understanding infectious disease transmission and persistence. Identifying and predicting transients across scales, from within-host to community-level patterns, plays an important role in combating ongoing epidemics and mitigating the risk of future outbreaks. Moreover, greater emphases on non-asymptotic processes will enable timely evaluations of wildlife and human diseases and lead to improved surveillance efforts, preventive responses, and intervention strategies. Here, we explore the contributions of transient analyses in recent models spanning the fields of epidemiology, movement ecology, and parasitology. In addition to their roles in predicting epidemic patterns and endemic outbreaks, we explore transients in the contexts of pathogen transmission, resistance, and avoidance at various scales of the ecological hierarchy. Examples illustrate how (i) transient movement dynamics at the individual host level can modify opportunities for transmission events over time; (ii) within-host energetic processes often lead to transient dynamics in immunity, pathogen load, and transmission potential; (iii) transient connectivity between discrete populations in response to environmental factors and outbreak dynamics can affect disease spread across spatial networks; and (iv) increasing species richness in a community can provide transient protection to individuals against infection. Ultimately, we suggest that transient analyses offer deeper insights and raise new, interdisciplinary questions for disease research, consequently broadening the applications of dynamical models for outbreak preparedness and management.

**Supplementary information:**

The online version contains supplementary material available at 10.1007/s12080-021-00514-w.

## Introduction

Since the pioneering work of Kermack and McKendrick (1927), a large proportion of epidemiological modeling has been based on analyses of deterministic, nonlinear dynamical systems (Guckenheimer and Holmes 1983; Strogatz 2018). The primary focus of such studies has been to identify the stability of *attractors*, the special solutions to which all trajectories eventually converge, e.g., equilibria, limit cycles, or chaotic attractors (Schwartz and Smith 1983; Diekmann and Heesterbeek 2000; Brauer et al. 2019). However, in the broader context of ecological systems, there has been a growing recognition that asymptotic dynamics of models give only a partial and potentially misleading impression of what we should expect to observe in the real world (Hastings et al. 2018). The dynamical characteristics of *transients*, trajectories that have *not* reached an attractor, may tell us more about real systems than can be gleaned from attractors. If transients play an important dynamical role in ecological systems, then it stands to reason that they deserve attention when assessing epidemics across human, wildlife, and agricultural systems and designing management strategies to combat them.

As an example, the general concept of transient disease dynamics has recently entered public consciousness in the ongoing COVID-19 pandemic. As of May 2021, more than three million deaths have been reported worldwide, accompanied by major disruptions to the global economy (McKibbin and Fernando 2020), transportation networks (e.g., travel bans, control of mass population movement; Chen et al. 2020), and social norms (e.g., physical distancing, household quarantine, closures of schools and university campuses; Wilder-Smith and Freedman 2020). In response, there have been numerous media headlines early on urging the public to help “flatten the curve”, a message promoting the idea that preventive actions can help reduce transmission and avoid overloading healthcare systems. In a world that continues to grapple with this crisis, transient analyses (e.g., Arthur et al. 2020; Ferguson et al. 2020) have assisted in forward projections of outbreak severity, estimations of resurgence risk, and policy recommendations ahead of worst-case scenarios.

**Fig. 1:**
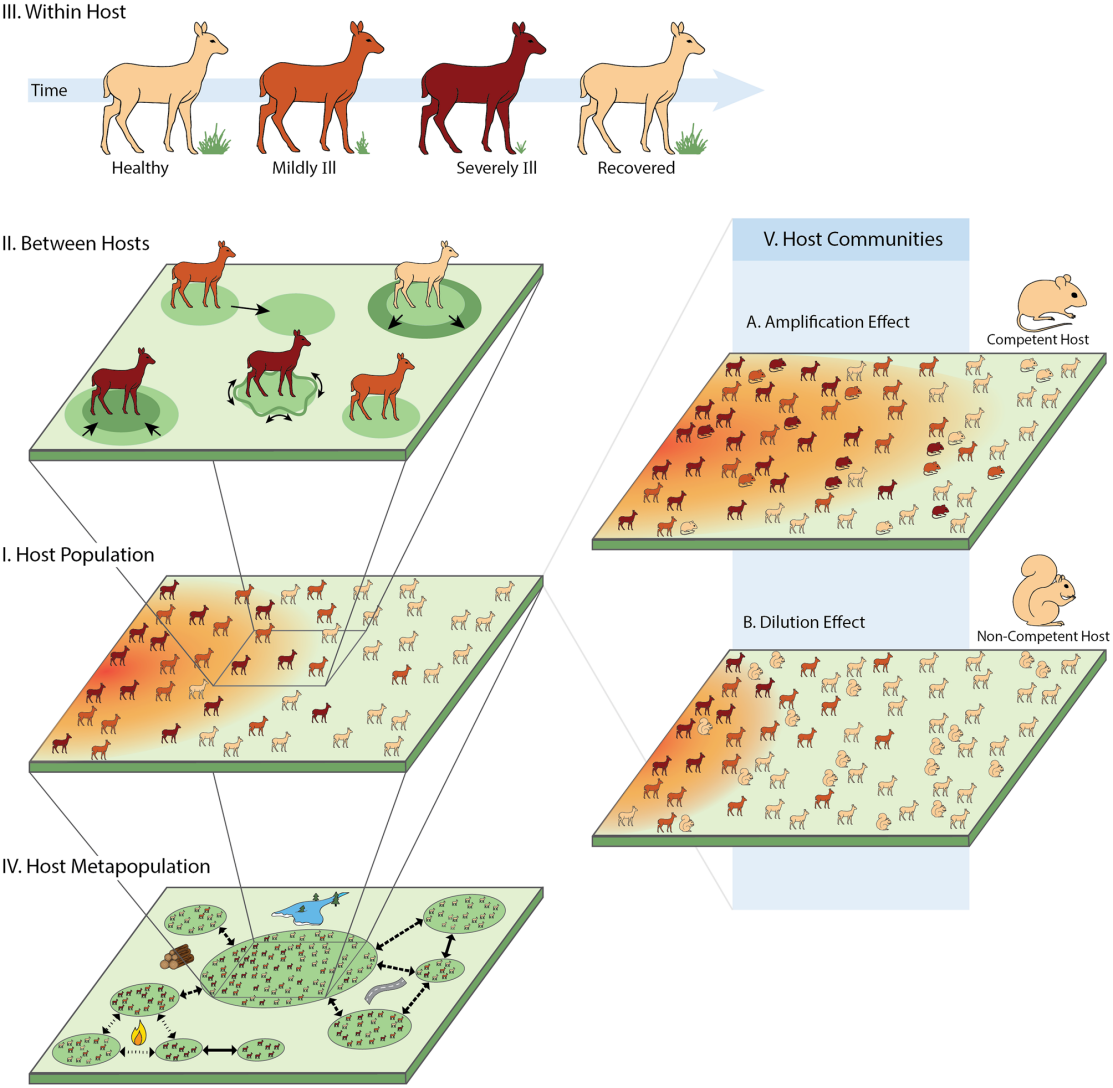
Examples of transient disease dynamics organized along an ecological hierarchy from within-host processes to host communities. Section III: Within an infected host, illness-induced anorexia can strongly influence infection dynamics prior to host recovery. Section II: Between hosts, transitional space use patterns before a home range or territory is stabilized can provide rare opportunities for infective contacts. Section I: At the host population level, the critical epidemiological questions address the spread of a transmissible pathogen through the population over time. Section IV: Within a host metapopulation, landscape disturbances (e.g., flooding, forest clearcutting, road construction, wildfires) may lead to transient windows of connectivity between habitat patches that reappear at various frequencies (solid lines: low; dashed lines: intermediate; dotted lines: high). Section V: At the host community level, interspecific variation in reservoir competence—shown here using examples for Lyme disease (see Levi et al. 2016)—can reduce the risk of infection to a particular host species, a phenomenon referred to as the dilution effect, or increase it as per the amplification effect. Roman numerals next to the ecological scales indicate corresponding sections in the text. Illustration credit: Life Science Studios (https://lifesciencestudios.com)

As COVID-19 information accumulates, so too has evidence of transient dynamics related to its pathogenesis, transmission window, and management efforts. Symptoms of some infected individuals under clinical care have been shown to subside temporarily before the sudden onset of severe illness (Zhou et al. 2020). SARS-CoV-2 emerged in the human population following a rare cross-species transmission event from a wildlife reservoir (Andersen et al. 2020; Wu et al. 2020). Cases were initially localized to the region where the virus spilled over into humans, individual movement and metapopulation connectivity drove subsequent transmissions and spatial spread across the globe (Wu and McGoogan 2020). As the outbreak spread, connections into and out of affected populations were transiently strengthened, suppressed, or redirected as a result of mass exodus, government-imposed lockdown, and self-quarantines (Jia et al. 2020). These short-lived patterns at the within-host, individual host, and host metapopulation levels show that, to fully understand the disease and control the pandemic effectively, it is not sufficient to only predict the system’s transient dynamics at the population level, e.g., measuring disease incidence. Instead, we must also account for transient *structural* changes that are reflected in lower- and higher-level processes (Fig. 1).

In part one of this paper, we examine the prevalence and properties of transients in observed time-series of infectious disease incidence and the analytical methods used to detect them. In subsequent parts, we draw from multiple disciplines transecting disease ecology, underlining timely case studies and theories that demonstrate different forms of structural transients.

We use case studies of both human and animal diseases to specifically show that at the individual host level, transient movement dynamics can modify opportunities for transmission events over time. We also show that within-host energetic processes can lead to transient dynamics in individual immunity, pathogen load, and transmission potential. In metapopulations, transient connectivity between discrete populations in response to environmental factors and outbreak dynamics can determine outbreak trajectory and pathogen spread across spatial networks. Finally, we show that increasing species richness in a community can provide transient protection to individuals against infection. Our survey of transient disease dynamics across ecological scales reinforces the idea that hierarchical factors affecting pathogen exposure and susceptibility to infection must be aligned in space and time to convert transmission risk to reality (Plowright et al. 2017).

## I. Transient behaviors in population models

The term “population” can be broadly applied to any collection of conspecifics that are not explicitly connected through a spatial network (for that description, see metapopulation level transients in Section IV). The transient nature of outbreak dynamics in a population can be observed across a variety of temporal scales determined by the duration of pathogen persistence.

### Common scales: regional to national, single epidemic duration

When a pathogen emerges or re-emerges, such as COVID-19 or Ebola, respectively, asymptotic dynamics are not the primary concern for disease management efforts. Instead, attention is focused on characterizing the initial pattern of spread, forecasting cases and deaths in the absence of interventions, and investigating (usually through simulations) whether and how the outbreak can be contained (Ferguson et al. 2016). Typically, there is a flurry of activity to estimate the basic reproduction number (Anderson et al. 1992; van den Driessche and Watmough 2002; Wallinga and Teunis 2004; Pourbohloul et al. 2009; Park et al. 2020), initial growth rate (Wallinga and Lipsitch 2007; Ma et al 2014; Champredon and Earn 2016; Chowell 2017; Earn et al. 2020), expected final epidemic size (Kermack and McKendrick 1927; Ma and Earn 2006), etc., and how the estimated values of these quantities should influence control strategies (Bauch et al. 2003; Earn et al. 2012; Molina and Earn 2015). In most models, the dynamics of interest are transient. Thus, in the context of forecasting and controlling outbreaks, whether for SARS (Lipsitch et al. 2003), pandemic influenza (Yang et al. 2009), Ebola (Bellan et al. 2014), Zika (Gao et al. 2016), COVID-19 (Anderson et al. 2020), or other diseases, there has always been an appreciation for, and emphasis on, transient dynamics.

The transients that span only a single epidemic are short-lived relative to any kind of asymptotic behavior. The timing, intensity, and targets of response during the transient phase can have drastic consequences for intervention success (e.g., Ferguson et al. 2001; Haydon et al. 2006; Tao et al. 2021). Recently, there has been an increased interest in dynamic control strategies that can adapt to the transient states of the epidemic (e.g., Handel et al. 2007; Probert et al. 2019) and resolve the epistemic uncertainties about transmission and treatment via real-time surveillance (Shea et al. 2014; Bradbury et al. 2017). These temporally nuanced approaches have typically led to significant improvement in management outcomes. As public health faces a growing threat from emerging infectious diseases (Jones et al. 2008; Heymann et al. 2015), new theories of dynamic control strategies that can provide timely, context-dependent solutions (e.g., when to reprioritize control objectives) may be better positioned to facilitate discourses between modelers, stakeholders, and policymakers.

In the age of social media, fears regarding the safety of recommended practices (e.g., vaccination, use of face masks) are prone to amplify general resistance to control strategies intended to lower susceptible recruitment and transmission opportunities. Recent game-theoretical models (e.g., Bauch and Earn 2004; Bauch and Bhattacharyya 2012) have explored individual vaccination behavior during bouts of vaccine scares. Additional theories that anticipate transients in the dynamics of both disease incidence and public trust could help manage policy expectations according to social currents and identify effective alternative strategies.

### Common scales: regional, decadal

**Fig. 2:**
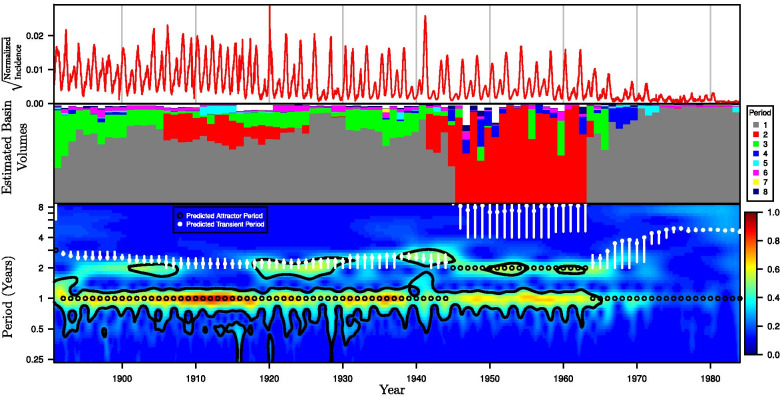
Comparison of predicted vs. observed measles dynamics in New York City (NYC), 1891–1984. Reprinted with permission from Hempel and Earn (2015). *Top panel*: Square root of measles case reports, normalized by total concurrent population. *Middle panel*: Approximate volumes of basins of attraction near the observed NYC measles incidence for each year from 1891 to 1984. For each year, we display the proportion of 10,000 simulations that reach each period. See Hempel and Earn (2015) for details. *Bottom panel*: Color depth plot of a continuous wavelet transform of the square root of normalized observed NYC measles cases (color warmth scales with spectral power and 95% significance contours are shown in black). The predicted attractor and transient periods are overlaid (only the periods of annual and biennial attractors are short enough to appear on this graph and to be observable). Unfilled black circles identify predicted attractor periods that are consistent with the observed data. White vertical bars show the possible ranges of the transient period associated with these attractors; the filled white dot on each bar marks the median transient period. There is a good qualitative agreement between the predicted transient periods and the observed power near periods 2 and 3 during 1891–1909, 1917–1945, and 1964–1973

In contrast to novel epidemics, for endemic diseases, the system’s dynamical behavior away from the attractor is generally maintained much longer. Transients could play a dominant role since temporal forcing and perturbations might keep orbits away from an attractor (Schwartz and Smith 1983; Keeling et al. 2001; Bharti et al. 2011; Becket et al. 2019). These types of dynamics are notably associated with oscillatory incidence time-series found in endemic childhood diseases (Fig. 2), in particular, diseases such as measles, rubella, and whooping cough that are strongly affected by contact differences between school holidays and school terms (London and Yorke 1973; Earn et al. 2000; Keeling et al. 2001; Bauch and Earn 2003). In early studies, identifying the periods of the system attractors alone correctly predicted the annual or biennial spectral peaks in the incidence time-series (Earn et al. 2000). However, the same approach does not capture the “non-resonant peaks” that occur at arbitrary frequencies and are sometimes found to be the most significant for the long-term dynamics for these diseases. Bauch and Earn (2003) identified noise-sustained transients to be the source of the non-resonant peaks. Their method of linear perturbation analysis involves converting a continuous-time model into a Poincaré map and then computing the eigenvalues of the linearization about its attractors. The results show that the greater presence of demographic noise associated with a smaller population can slow or prevent convergence to the attractor. Reduction of susceptible recruitment level over time (i.e., fewer births or more vaccination) can also promote transient effects for (at least) two reasons: lower effective reproduction number yields (i) more prominent non-resonant peaks associated with non-convergence to attractors (e.g., Fig. 2c of Bauch and Earn 2003) and (ii) more likely stochastic switching between basins of co-existing attractors (Earn et al. 2000; Hempel and Earn 2015).

Although non-resonant spectral peaks in incidence time-series have been identified largely in the context of human diseases, similar dynamical patterns can also be expected to unfold in temporally forced zoonotic diseases. For instance, widespread outbreaks of Rift Valley fever, an endemic, arthropod-borne disease that affects both human and ruminant populations, typically occur in cycles varying between 7 and 11 years (Manore and Beechler 2015). How the pathogen is able to persist during the inter-epidemic periods remains unclear. Recent model explanations have been limited to vertical transmission, seasonality of vector abundance, and environmental stochasticity (e.g., Cavalerie et al. 2015; Manore and Beechler 2015). Extending perturbation analyses to such systems might offer a new, mechanistically simpler explanation: much like the rubella dynamics in the pre-vaccine era (cf. Bauch and Earn 2003, Figs. 1c,g and 3), these infrequent large outbreaks, with frequent small outbreaks or no detectable outbreaks in between, could be no more than a manifestation of intrinsically driven non-resonant spectral peaks. This line of investigation might therefore help to better inform the surveillance programs for zoonotic pathogens that have potential for long-term, silent circulation.

**Fig. 3:**
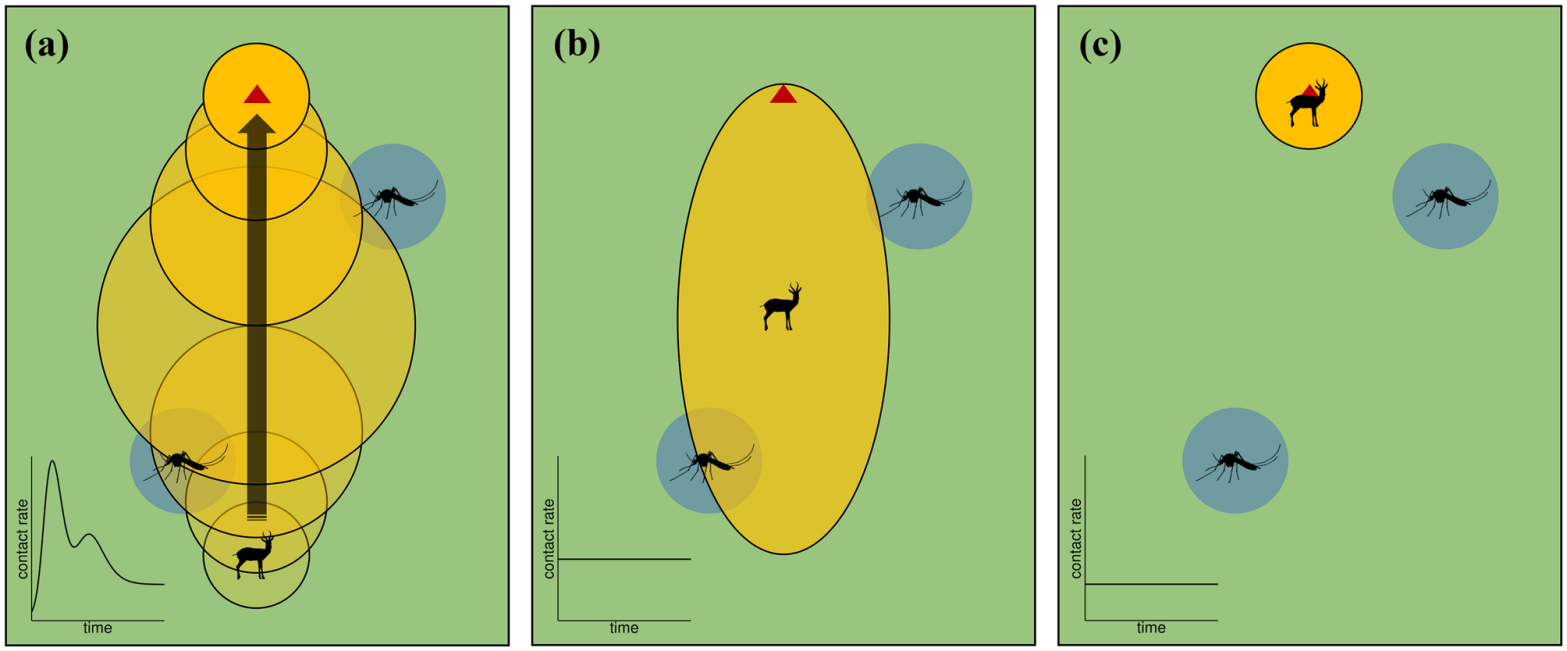
Contrasting model descriptions of a traveling host (or host group behaving as a unit) coming into contact with vector clusters in a constant environment. A ruminant herd is depicted to encounter mosquitoes when moving near watering holes, a key transmission pathway of Rift Valley fever (Manore and Beechler 2015). To account for transient space use dynamics (**a**), the movement of the host was modeled as a utilization distribution (UD) converging toward an equilibrium (yellow circles of increasing opaqueness) centered around a spatial point attractor (triangle). Here, time evolution of the UD is determined by a Fokker–Planck equation with constant coefficients in the advection and diffusion terms. Infective contact occurs at a rate proportional to the instantaneous overlap between the UD and the vector distribution (blue circles). See Fieberg and Kochanny (2005) for candidate indices of overlap, e.g., integral of the joint distribution. The resultant time-series of contact rate is characterized by irregular peaks. In **b**, the transient UDs are aggregated to produce a time-independent probability density surface of the host’s location, yielding a constant contact rate. The model in **c** incorporates only the asymptotic UD. This reduces the contact process to a constant, which could drastically underestimate the animal’s risk of infection over the course of its resettlement

### Transient structural dynamics in disease systems at sub-population levels

Despite the extensive use of population models, there remain epidemiological challenges where the needs to reduce uncertainties are better addressed by anticipating the potential experience of *individuals* at various stages of infection than by predicting the population dynamics. For instance, targeted conservation may require close surveillance of individual host movement near a small number of colonies. Clinical outcomes could also be improved by personalizing treatment based on the infection dynamics within each patient. In models suited for these purposes, the spatial scale of transient disease dynamics ranges from the host body size to the parts of an environment in which a host (or a group of hosts behaving as a single unit) may traverse; the relevant time scales tend to be short compared to those in population models.

## II. Effects of transient host movement dynamics

### Common scales: individuals to collective groups, short-term encounters to long-term range shifts

Within a population, the risk of pathogen transmission to a recipient host is largely driven by host movement (Fig. 1, Section II). Direct transmission occurs through contact between a susceptible and an infected host while indirect transmission can occur when an infected and susceptible host spatially overlap with some temporal lag (Dougherty et al. 2018). Advanced knowledge of host movement contributes to our understanding and management of emerging zoonotic diseases. Numerous studies have highlighted the downstream effects of myriad movement behaviors including bat foraging activities during periods of agricultural and urban intensification (e.g., Nipah virus: Pulliam et al. 2012, Hendra virus: Plowright et al. 2011), long-distance dispersals of pre-breeding ruminants (e.g., bovine tuberculosis, Rift Valley fever: Caron et al. 2016), and worker resettlements and air travel (e.g., Ebola: Alexander et al. 2015, Zika virus: Ali et al. 2017). Despite the importance of host movement as a predictor of outbreaks, many compartmental models assume populations are well-mixed and the per-capita contact rate is time-invariant in the absence of temporal forcing. These simplifying conditions imply homogeneity in population contact structures and that each host has a stationary range distribution unless changes happen in the environment (e.g., seasonal transitions). By not recognizing contact rate as a host-specific variable that is dependent on both space and time, important transmission opportunities could be missed.

Mechanistic space use models (see a historical overview in Potts and Lewis 2014) have laid the foundation for predicting movement dynamics of individual hosts. The analyses predominantly focus on the utilization distribution (UD), a probability density surface that describes the range and uncertainties of an individual’s possible locations at any point in time. UDs can be solved numerically when they are modeled using partial differential equations or estimated from outputs of agent-based simulations (see Potts and Lewis 2014 and references therein). A host’s UD may equilibrate slowly, if at all (Fryxell et al. 2008; Potts and Lewis 2016). It may also be sensitive to local conditions, such as terrain (Moorcroft et al. 2006), resource availability (Moorcroft et al. 2006; Bateman et al. 2015), and the presence of conspecifics or predators (Lewis and Moorcroft 2001; Bateman et al. 2015; Tao et al. 2016; Potts et al. 2018). The transitional UDs may be highly variable and create short-term opportunities for host-pathogen contacts that impact population, metapopulation, and community level transmissions (Plowright et al. 2011; Ramsey et al. 2014; White et al. 2020). Conversely, an individual’s exposure to pathogens could temporarily change where it may go (Zidon et al. 2017). Thus, explicit inclusion of transient movement dynamics in disease models could have far-reaching consequences for the implementation of biosurveillance systems.

In basic compartmental models, transmission is commonly treated as a single process: a product of infective contact rate, averaged over both time and individuals, and the probability of transmission during contact. We propose separating these two components and describing the encounter process as a function ${f}_{c}$ of the transient space use pattern at the host level, such that, without vital dynamics,

1 $$\dot{S}=-\kappa \sum_{i=1}^{S}{f}_{c}\left({u}_{i}\left(\mathbf{x}, t\right), \sum_{j=1}^{\widehat{I}}{v}_{j}\left(\mathbf{x},t\right)\right),$$

where ${u}_{i}(\mathbf{x},t)$ and ${v}_{j}(\mathbf{x},t)$ denote the UD of each susceptible host and the distributional pattern of each pathogen source, respectively. $S$ is the number of susceptible hosts. $\widehat{I}$ may refer to the number of (a) infected hosts, (b) vector-infested sites, or (c) patches containing pathogen. Upon contact, transmission happens with probability $\kappa$. Epidemic predictions that account for transient space use dynamics can differ considerably from those based on asymptotic, mean field approaches (Tao et al. 2018). We conjecture that, by tracking, at minimum, ${u}_{i}\left(\mathbf{x},t\right)$ and the consequent rate of contact over time, this framework will help bring into focus the period and spatial region of host movement most critical for disease transmission (Fig. 3).

Mass vaccination campaigns in regions with underperforming health services often require mobilization (e.g., house-to-house visits) of healthcare workers for vaccine delivery (polio: Curry et al. 2014; measles: Mbabazi et al. 2015), reaching inhabitants in a limited geographical region at a time. In such scenarios, the deployment of response personnel into a target population can also be modeled as a UD evolving over time. Evaluating the impact of this strategy on management success allows us to address operational questions, namely, how fast or from where response effort should be distributed, thus extending control recommendations beyond the equilibrium criterion (e.g., threshold herd immunity). Tao et al. (2018) did so by combining a diffusion model of probabilistic vaccine delivery with an agent-based simulation of disease transmission. This produces site-specific, time-dependent rates of vaccine uptake. The race between local vaccinations and disease spread determines the optimal deployment strategy to minimize overall outbreak severity. We expect to see further development of models centered on transient phases of human movement that can be used to optimize resource allocation and minimize delays during an emergency response.

## III. Effects of transient within-host dynamics

### Common scales: pathogen population inside a host, time to recovery or death

Host level movement processes that drive pathogen transmission are often tightly coupled to environments *within* hosts: a host’s immunity against invading pathogens weaken following energetically costly migration (Owen and Moore 2008); individuals carrying high pathogen loads may also become sedentary and shed pathogens in concentrated areas that are frequently visited by conspecifics (Patterson and Ruckstuhl 2013). Modeling infection dynamics of individual hosts could therefore help us understand the epidemiological costs of certain host behaviors (Lunn et al. 2019) and identify short-term ecological contexts that might promote the existence of superspreaders (Hawley and Altizer 2011).

Infection dynamics arise from within-host interactions between host immunity and pathogens, which behave, respectively, as predators and prey that also compete over limited resources (Smith and Holt 1996; Cressler et al. 2014; Greenspoon et al. 2018). The onset and duration of specific immune phases in an infected host, and the associated shifts in its metabolic processes, can regulate infection dynamics, affecting the time until pathogen clearance, i.e., host recovery from infection. Dynamic models have been recently developed to track energetic flows within hosts. The included mechanisms may describe energy (or resources) being assimilated after feeding, stolen by pathogens, and allocated toward host immunity, maintenance, reproduction, and growth (see Hall et al. 2009; Hite and Cressler 2018; Civitello et al. 2018; Van Leeuwen et al. 2019). The model framework has helped explain divergences in disease chronicity, i.e., why some hosts clear a pathogen rapidly (acute infection) while others develop persistent, sometimes life-long, infection (chronic infection). For instance, Van Leeuwen et al. (2019) demonstrate how a high pathogen dose can strongly modulate a host’s metabolism and lead to long-term pathogen persistence, as opposed to a low dose that could be quickly cleared. Transient analysis may thus resolve well-documented uncertainties in host pathologies (see Wilber et al. 2017) and project individual risks of onward transmission.

Although immune responses are often energetically expensive, many infected individuals have been found to voluntarily suppress their feeding behavior in order to “starve out” the pathogen (Fig. 1, Section III). Illness-mediated anorexia (reviewed in Hite et al. 2020), a temporary but substantial suppression of appetite during key phases of infection, can function as a first line of defense by limiting pathogen growth and replication (“antigrowth resistance”) or infection-induced pathology (“tolerance”). This caloric restriction begins after seconds to days of exposure and appears strategically tuned to specific stages of pathogen life cycles. To further complicate matters, empirical evidence indicates that anorexia can be manipulated, directly or indirectly, by pathogens to increase their rate of shedding or vector transmission (Adamo et al. 2007; Rogers and Bates 2007; Rao et al. 2017). There is currently only a small number of studies that model the interactions between infection dynamics and anorexia (see Hite and Cressler 2019; Hite et al. 2020). Nevertheless, subverting the loss of appetite has been a part of many standard treatments for humans and livestock (Fox et al. 2002; Schütz et al. 2014; Rodrigues et al. 2015; Hite et al. 2020). Future theoretical advances in this area could help evaluate their efficacies across different individuals and optimize treatment schedules to minimize infection duration.

Models of within-host dynamics have mostly been used to predict population-level consequences such as infection prevalence and evolution of virulence. Typically, the pathogen is assumed to reach carrying capacity immediately after successful host invasion and colonization, irrespective of local metabolic condition (Mideo et al. 2008). Resource availability outside the host is sometimes incorporated as a slowly changing external factor. This time-scale separation has aided mathematical tractability and provided general insights into pathogenesis, transmission fitness, and unwelcome repercussions of vector-reduction programs (Mideo et al. 2008; Handel and Rohani 2015; Civitello et al. 2018). However, an alternative framework could integrate multiple intrinsic time scales: i.e., slow environmental resource dynamics interacting with fast infection dynamics. Models that “internalize” the slowly changing variables might reveal long, relatively disease-free periods in individuals interrupted by infection during population-wide epidemics (see Hastings et al. 2018 on how slow-fast systems promote long-lived, quasi-stable transients).

### Transient structural dynamics in disease systems above the host population level

In metapopulations and communities, inter-population and interspecific interactions drive transient disease dynamics (Fig. 1, Sections IV and V). At this upper echelon of the ecological hierarchy, transient behaviors typically operate over an area sufficiently large to accommodate substantial heterogeneity in contact structure, disease susceptibility, and pathogen transmissibility. Unlike population models, the time scale can be a fraction of an epidemic cycle (or a natural disturbance cycle); alternatively, it can persist for many host generations until significant species turnover has been achieved.

## IV. Effects of transient network dynamics in host metapopulation

### Common scales: multiple populations, phase of an outbreak

Since Hanski’s classic works on the ecological importance of metapopulation structure (e.g., Hanski 1998), the notion of connectivity between geographic areas (e.g., habitat patches) has been the cornerstone of network-related models, including the many theoretical frameworks currently available for predicting disease spread. In metapopulation models, transient dynamics related to local and global population densities have long been a subject of interest (Saravia et al. 2000; Ovaskainen and Hanski 2002; Labra et al. 2003); nevertheless, connectivity itself, i.e., the spatial distribution of population flow rates, is typically assumed to exist in an asymptotic state (but see Perry and Lee 2019; Karnatak and Wollrab 2020). Given their enormous implications for transmission dynamics (Hess 1996; Grenfell and Harwood 1997; Hanski 1998; Keeling et al. 2004), models that can explicitly account for the transient dynamics of connectivity can help quickly predict and prevent the spread of infectious diseases. Below, we outline some of the most common types of transient dynamics of connectivity pertinent to disease modeling. These are classified as network advection, suppression, and diffusion (Supplementary Table).

**Fig. 4:**
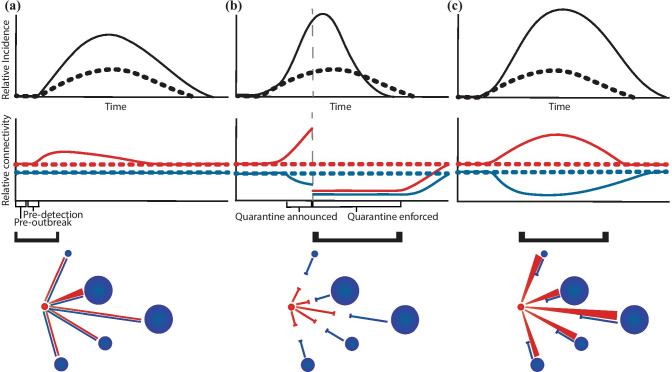
Examples of outbreak-induced transients in human network connectivity: **a** network advection: health-seeking behavior; **b** network suppression: disease avoidance behavior; **c** network diffusion: disease escape and avoidance. *Top row*: Metapopulation level incidence time-series with (black solid) and without (black dashed) the transient population flows pictured in the bottom schematic. *Middle row*: Metapopulation level connectivity time-series between an infected patch (red circle below) and its neighboring uninfected patches (blue circles). Red lines show network connectivity in the presence (solid) and absence (dashed) of the illustrated transient population flows out of the focal infected patch; blue lines show it in the presence (solid) and absence (dashed) of transient flows into the focal infected patch. *Bottom row*: Transient population flows between patches (circles). Patch size is proportional to local population size. Straight lines show unchanged connectivity dynamics; triangles show increased flow from point to wide end; bar lines show no immigration. Brackets above the schematic mark the time window during an outbreak when these transient dynamics might occur

Network advection (Fig. 4a) denotes transient immigration to a patch. This can describe safety or health-seeking behavior in humans, such as when patients travel to areas with more stability or higher quality, more accessible health resources (Bharti et al. 2015). We observed this trend during the 2013 Ebola outbreak in West Africa (Bogoch et al. 2015; Fallah et al. 2018), which was facilitated by improvements in transportation infrastructure relative to previous sites of Ebola spillover (Chowell et al. 2004; Malvy et al. 2019). Advection may also describe species range shifts. Marsh fritillary butterflies may relocate en masse following land cover changes (e.g., forest clearcutting) that result in the conversion of an inhospitable matrix habitat into a transient movement corridor (see Wahlberg et al. 2002; Driscoll et al. 2013). In both scenarios, the short-term establishment of directional bias in connectivity, especially early in an outbreak, may allow pathogen dispersal into and colonization of new, susceptible host populations (Helble 2011; Alegana et al. 2012).

Under network advection, the potential impact of an outbreak would be strongly dependent on travel time relative to the mean infectious period. Direct and rapid travel toward health care while infectious is essential for timely treatment and recovery of an individual; yet, it also enables onward transmission at the destination patch. Conversely, circuitous routing that delays travel time past the infectious stage may increase the chance of pathogen introduction at stopover sites but prevent introduction at the destination. Therefore, differences in transportation infrastructures and habitat matrix conditions (terrain, elevation, favorability, and other attributes affecting crossings) might have long-term, opposing effects on disease dynamics that should be explicitly considered in multi-patch models.

When new infections are concentrated in one or a few patches, severing connections between infected and uninfected or low-prevalence populations, i.e., network suppression (Fig. 4b), becomes critical for containment (Chinazzi et al. 2020; Wells et al. 2020). Suppression may arise intrinsically through disease avoidance behavior (Widmar et al. 2017), or be a result of top-down quarantine measures (SARS: Riley et al. 2003, COVID-19: Ainslie et al. 2020; Chinazzi et al. 2020). In wildlife systems, transient habitat fragmentation caused by extreme weather events can produce an analogous effect. For example, after a riverine floodplain has experienced unusually high precipitation, terrestrial species may be temporarily unable to move between habitat patches due to localized flooding (e.g., Angelone et al. 2011; Erös and Grant 2015).

There are significant geographic differences in compliance with prevention guidelines across socio-economic and urban-rural divides (see Painter and Qiu 2020; Wright et al. 2020), particularly if polarization in attitude towards public health measures grows. Future multi-patch epidemic models might need to investigate the correlation between local compliance and the duration and spatial scale of network suppression. On the other hand, to study the effects of suppression on epizootics, metapopulation models may consider incorporating sudden appearances of impermeable matrices and their restorations to suitable habitats over time (see the concept of transient connectivity window in Ziegler and Fagan 2014).

As incidence within a patch surges, resident individuals might attempt to escape toward disease-free patches until the outbreak is brought under control. Meanwhile, the immigration rate to infected patches should decline due to disease avoidance or restrictions on patch entries (Médecins Sans Frontières 2009; Bengtsson et al. 2015). Co-occurrence of these two transient responses, termed network diffusion (Fig. 4c), has been observed in management scenarios characterized by “leaky lockdowns” (Ebola: Dellicour et al. 2018; Mukandavire et al. 2011, cholera: Bengtsson et al. 2015). Diffusion can also stem from sporadic, patchy disturbances. Low-intensity wildfires, for instance, can induce local wildlife to search for suitable habitats beyond the fire boundary (Nimmo et al. 2019). This temporary change in connectivity, which could disseminate pathogens via infected individuals into neighboring populations within a short time interval, promotes the emergence of an outbreak cluster at the metapopulation level.

Network diffusion aptly describes the Syrian refugee crisis, where large cohorts with low levels of immunity against vaccine-preventable diseases (e.g., polio, measles, rubella) entered new environments, increasing the transmission risk in refugee camps as well as in the imperfectly immunized communities to which they were displaced (Eichner and Brockmann 2013). We postulate that synchronous pathogen invasions into multitudes of new susceptible pools, in responses to humanitarian crises or stochastic habitat losses, are widespread across human and wildlife systems. Models that can capture the effects of such events could be critical for predicting epidemiological repercussions of socio-political unrest and human-accelerated environmental changes.

## V. Transient effects of host community dynamics

### Common scales: ecosystem, multiple host generations

The idea that increased species richness reduces disease transmission, referred to as the “dilution effect” (Keesing et al. 2010), is popular yet controversial. This hypothesis posits that adding infection-incompetent host species, or species that are incapable of efficient onward pathogen transmission, to a community will increase the density of pathogen sinks and thus *reduce* the spread of a host-specific pathogen (Fig. 1, Section V). Therefore, in a diverse community composed of relatively abundant incompetent host species, a susceptible, competent host individual would have a lower risk of infection by benefiting from “safety in numbers” (Johnson et al. 2013).

Negative correlations between host diversity and infection rate have been broadly found in recent studies, leading to suggestions of its generality (Keesing et al. 2010; Civitello et al. 2015). However, large-scale studies (e.g., Dunn et al. 2010; Randolph and Dobson 2012; Lafferty and Wood 2013; Wood et al. 2017) tend to indicate an opposite trend, with evidence of biodiversity *increasing* disease transmission, presumably due to an amplification effect characterized by a disproportionate abundance of competent host species for a generalist pathogen (Keesing et al. 2010). These contradictory patterns may be explained by the dilution effect being a scale-dependent phenomenon that is observed either in small-scale experiments or under short durations (see Cohen et al. 2016; Halliday and Rohr 2019; Rohr et al. 2019). The latter explanation considers that added hosts, irrespective of their competence, might buffer the original hosts against infections early on by intercepting free-living stages of the pathogen (e.g., parasite eggs, cysts). However, if the added host is competent, the initially observed dilution effect will weaken over time and the original hosts could experience a net increase of infection risk after the pathogen spreads through the newcomers. In other words, amplification can transiently appear as dilution in the short term. The same principle may apply to human communities. For example, immigration of many vaccine-immunized hosts into a susceptible population may provide it with transient herd immunity via a temporary increase in immune diversity. However, the risk of infection will be amplified in the long run (due to high population density) if immunity fades or if new offspring are not sufficiently vaccinated.

We present a heuristic model to demonstrate how infection risk changes over time within a diversifying community. It assumes that a new host species is seeded randomly in a well-mixed system that previously contains a single competent host species. The pathogen’s questing, attacking, and consuming stages are combined using a separation of time scales, giving a saturating transmission function that is necessary for dilution to occur (after Lafferty et al. 2015). We further assume the transition from infection to pathogen production is fast relative to host generation time. In this system,

2 $$\dot{I} =\frac{b\beta {(X}_{1}-I)I}{d+\beta {X}_{1}}-\gamma I$$

where $I$ is the density of infected individuals of any host species and ${X}_{1}$ is the density of all individuals of target host species 1. Free-living pathogen stages are produced at rate $b$ per infected host and removed at rate $d$. Contact with any host (susceptible, infected, or non-competent) occurs at rate $\beta$ and leads to the loss of the pathogen stage. A new infection is generated if the host is uninfected and competent. To maintain constant host density, infected hosts are replaced with susceptibles at rate $\gamma$ upon death or recovery. When species 1 is the only host present, ${X}_{1}-I$ is the density of susceptible hosts, and this model would reduce to the familiar $SI$ model if one assumes that the pathogen stage has such a high background morality rate that loss due to contact is trivial.

**Fig. 5:**
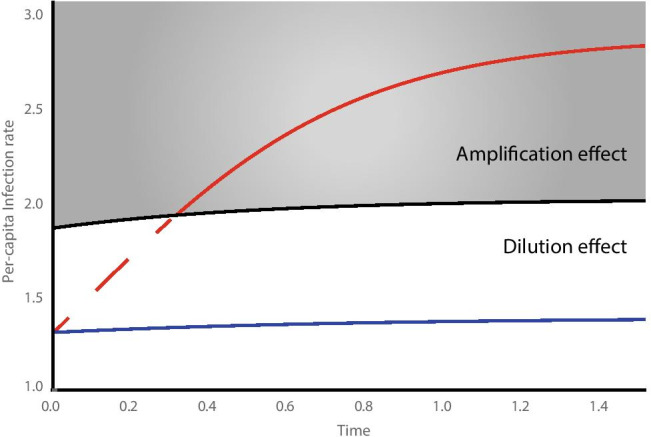
Per-capita infection rate of a target host species over time under three biodiversity scenarios. The addition of a non-competent host in a single-species community leads to a dilution effect (white space), characterized by the target host species being infected at a permanently reduced rate (cf. blue and black lines). In contrast, adding a competent host results in only a temporary rate reduction (dashed line), reflecting a transient dilution effect. As the system approaches its new attractor, the target host species becomes infected at a higher rate than it would without the increase in species richness (cf. red and black lines), in accordance with an amplification effect (gray space). Model parameters ${X}_{1}$, ${X}_{2}$, and ${X}_{3}=1$, $K=1.2$, $\gamma =0.1$, $b=5$

Adding a competent host species ${X}_{2}$ at the system equilibrium modifies the time derivative to

3 $$\dot{I} =\frac{b{(X}_{1}+{X}_{2}-I)I}{K+{X}_{1}+{X}_{2}}-\gamma I$$

where $K=d/\beta$ is the familiar Michaelis-Menton half-saturation constant from enzyme kinetics (Lafferty et al. 2015). A simple derivation shows that, initially, the per-capita infection rate for host species 1$,$

4 $${\Omega }_{1}\left(t=0\right)=\frac{{bX}_{1}-\gamma \left(K+{X}_{1}\right)}{K+{X}_{1}+{X}_{2}}$$

decreases with the addition of another competent host species, much in resemblance to the dilution effect (Fig. 5). However, as $t\to \infty$, the rate

5 $${\Omega }_{1}^{*}=\frac{b{(X}_{1}+{X}_{2})}{K+{X}_{1}+{X}_{2}}-\gamma$$

exceeds the single-species threshold. In contrast, adding a non-competent host species ${X}_{3}$ always reduces the per-capita infection rate for the target host,

6 $${\Omega }_{1}^{*}=\frac{{bX}_{1}}{K+{X}_{1}+{X}_{3}}-\gamma$$

even as the system heads to a new equilibrium (Fig. 5).

In summary, at the initiation of a study where a second host species is introduced, both diluting and amplifying hosts transiently appear to dilute transmission. Over time, however, their variable levels of competence lead to divergent epidemic potentials. In practice, an amplification effect could be very difficult to detect from field and experimental data collected over short time spans. We therefore caution that the support for a dilution effect by these studies should not be extrapolated to predict long-term system behavior, or be generalized. Moreover, the existence of transients in our model results suggests that disease-control strategies with immediate benefits may have future negative consequences that should be carefully considered.

## Concluding remarks

Analyses of transients in dynamical systems are of vital importance to disease research. Although the scientific interest in transients was first motivated by rigorous mathematics, the results of many recent models carry implications for actionable measures that are far from theoretical abstractions. Predictions of transient dynamics, with a prevailing emphasis on an “ecologically relevant time scale” (Hastings 2004), increasingly demonstrate their wide-ranging utilities to problems of applied epidemiology. Here, using a sample of models across ecological scales, we highlight the importance of understanding transient dynamics at different stages of disease transmission and outbreak response. This approach can improve standard protocols of drug delivery, timeliness of outbreak surveillance and intervention, accuracy of epidemic forecast, and effectiveness of large-scale disease management policies.

We observed that transient patterns at a population level may erupt early and rapidly, e.g., during an outbreak, or may reoccur periodically as in the case of many endemic childhood diseases. Individually, spatial interactions between a host and its environment through movement determine infective contact opportunities and the window of transmission; transient immune and physiological responses could mean the difference between recovery and chronic illness. At the metapopulation level, transient increase or loss of connectivity between patches may significantly reshape an epidemic curve. Within a host community, observations of a dilution effect can mislead conclusions regarding a species’ long-term infection risk.

We discussed a wide range of disease models to show transient behaviors may be critical for a variety of reasons. The importance of studying transient behavior depends on how long the system remains away from the attractors and how information collected during those periods might be interpreted and applied in an epidemiological context. For instance, the transients discussed in population and within-host models (Sections I and III) share the common feature that the asymptotic state can be disease-free. The adverse effects on individuals or populations occur during the transient phase in such cases; an ability to predict transients is advantageous for clinical care and outbreak management no matter how short the transient phase. This stands in contrast to the transient dynamics that are slow to equilibrate, which may describe endemic incidence, animal space use patterns, as well as conspecific rates of infection in a diversified community (Sections I, II, and V). The dynamics likely persist over the time scales that are most relevant for addressing questions in epidemiology. In the special case where the transient regimes are quasi-stable for a long time (see Hastings et al. 2018; Morozov et al. 2020), their short-term dynamics could be mistaken for an asymptotic state, which would undermine long-term forecasting (e.g., mislead conclusions on the generality of the dilution effect) and scenario planning. We also featured systems in which opposing inferences are drawn from the transient and the asymptotic behaviors (e.g., dilution versus amplification effects; Section V), and systems that contain multiple attractors with divergent outcomes (e.g., host recovery versus chronic infection; Section III). Here, the need for understanding transient dynamics is tied to the importance of early interventions that aim to maintain a system in, or force it towards, a more desirable state. Finally, in models of individual host movement and host metapopulations (Sections II and IV), the transient behaviors, which can be fleeting, reflect changes in the probabilities of rare, brief, or localized events (e.g., spillover infections, emergency travel restrictions) that might have long-term, large-scale implications for disease spread. Transient analyses of these systems could therefore be critical to developing early detection methods that are able to forestall low-risk but high-impact transmissions.

Recognizing the impacts of transient disease dynamics can help modelers decide which processes should be included in a system. At sub-population levels (Sections II and III), scheduled pharmaceutical interventions, territorial conflicts, and other events that delay equilibration tend to occur on a time scale shorter than the transient’s lifetime in the absence of these perturbations; as a result, the predicted transient phase could be substantially shortened by excluding them. By contrast, if the transient behavior were to dampen quickly such that the system’s controlling parameters stay near-constant during the transient’s lifetime, incorporating external processes might still be important in long-term studies since it may identify repeated transitions between the different transient and asymptotic states. In population and metapopulation models where changes in management and environmental conditions are often infrequent (Sections I and IV), predicting the timing of their recurrences could help evaluate multi-generational trends and sudden shifts in large-scale dynamics, thereby providing a stronger test of the system’s epidemiological resilience. Finally, in the two systems driven by spatial behaviors (Sections II and IV), the transients (e.g., individual migrations away from pathogen hotspots, changes in population flow patterns) are direct responses to the disease itself. A model that couples movement and disease dynamics may then contribute to the development of control strategies that are robust to behavioral changes by anticipating how hosts might balance the trade-offs between infection risk and travel cost as an outbreak continues.

Transients do not always lend themselves easily to examinations by conventional mathematical methods. Nevertheless, ongoing advances in dynamical systems analysis, numerical simulations, and network analysis show that transient dynamics can be modeled explicitly and combined with asymptotic analysis to enhance responses to infections, contagion, and outbreaks. The recent proposal of applying adaptive management approaches to guide public health decision-making (Shea et al. 2014) further suggests that our description of a disease system and the recommended management actions are transient in themselves due to inherent model uncertainty, which can be resolved over time through continuous surveillance. Consequently, a greater acknowledgment of transients will not only influence our model conclusions, but will also provide an objective basis for real-time model improvement.

## Supplementary Information

Below is the link to the electronic supplementary material.

Supplementary file1 (XLSX 11 KB)

## Data Availability

Epidemiological data used in the present manuscript are available at http://iidda.mcmaster.ca.

## Code availability

Software and custom code used in the present manuscript are available upon request.
